# Internalized Stigmatization, Social Support, and Individual Mental Health Problems in the Public Health Crisis

**DOI:** 10.3390/ijerph17124507

**Published:** 2020-06-23

**Authors:** Jiannan Li, Wenqi Liang, Bocong Yuan, Guojun Zeng

**Affiliations:** 1 International School of Business & Finance, Sun Yat-sen University, Guangzhou 510275, China; lijnanna@mail.sysu.edu.cn; 2 School of Tourism Management, Sun Yat-sen University, Guangzhou 510275, China; liangwq28@mail2.sysu.edu.cn

**Keywords:** internalized stigmatization, social support, mental health problems

## Abstract

This study investigates the relationship between internalized stigmatization brought on by epicenter travel experiences and mental health problems (including anxiety, depression, and shame) during the period of the novel coronavirus disease emergency in China. The cross-sectional data were collected using the time-lag design to avoid the common method bias as much as possible. Regression results using structural equation modeling show that the internalized stigmatization of epicenter travel experiences may have positive relationships with mental health problems (i.e., anxiety, depression, and shame), and such relationships can be moderated by social support. Specifically, the positive relationships between internalized stigmatization and mental health problems are buffered/strengthened when social support is at a high/low level. The findings of this study suggest that, in this epidemic, people who have epicenter travel experience could be affected by internalized stigmatization, no matter whether they have ever got infected.

## 1. Introduction

The novel coronavirus disease first reported in late December 2019, followed by an outbreak across Hubei Province, China, was later declared by the World Health Organization as a global public health emergency [1]. Many people had traveled in the epidemic area (for business or leisure purposes) before the outbreak. These people did not necessarily get infected. However, after they returned to their places of residence, and being at a high risk of infection, they were more likely to suffer internalized stigmatization associated with the travel experiences. The harmful effects of internalized stigmatization may work not only through the visible effects of discrimination by others, but also through the internal perceptions, beliefs, and emotions of the stigmatized person [2]. The travelers who came back from the epicenter may have the internal belief that they will be discriminated against or doubted by their community due to the stigma of “having once been to the epicenter”. Moreover, internalized stigmatization is found to hinder the recovery of mental health by eroding psychological resources [3]. Some research on infectious disease-related internalized stigmatization and mental health problems has reported that infectious disease-related internalized stigmatization is one of the important determinants of psychological problems among men who have sex with men [4] and among people living with HIV [5]. Accordingly, for people who have travel experiences in the epicenter, their internal fear of discrimination and exclusion may create negative emotions and cognition of self-deprecation, feeling dirty, and self-blame, making them suffer a series of mental health problems as a result.

Furthermore, social support, manifested as psychological and material resources from one’s social network (such as family members, friends, close partners) [6], can benefit one’s ability to develop positive self-awareness and to reduce negative emotions [7]. The high level of social support may be helpful to people who have travel experiences in the epicenter by providing them with more resources (such as emotional support and material resources) to regulate negative emotions and cognition caused by internalized stigmatization. Thus, social support may moderate the relationship between internalized stigmatization associated with epicenter travel experiences and mental health problems such that the negative effect of internalized stigmatization on mental health problems can be mitigated when the level of social support is high.

This study aimed to use cross-sectional data to empirically investigate the relationship between internalized stigmatization brought on by epicenter travel experiences and mental health problems (including anxiety, depression, and shame) and to reveal the mechanism of social support with respect to the above during the period of the novel coronavirus disease emergency in China. This research question is not only of clinical and policy significance [8], but it also enriches research about infectious disease-related internalized stigmatization. First, this pandemic has affected more and more areas, which calls for urgent focus. Second, previous studies focused on the patients themselves. In contrast, the internalized stigmatization of people with epicenter travel experience during the pandemic has rarely been investigated. The population we investigated were those who did not necessarily get infected. Finally, prior studies mainly focused on the effect of attributed stigmatization (perception of how most people feel) on mental health problems. This study, on the other hand, explored the effect of internalized stigmatization (how respondents themselves feel) on mental health problems, and further revealed the moderation mechanism underlying such a relationship.

## 2. Literature Review

### 2.1. The Impact of Internalized Stigmatization on Anxiety

Anxiety is a psychological response to external stressors, which refers to an emotional experience of worrying and uneasiness about something that has happened or is about to happen. It is marked by psychological symptoms such as excessive worry and apprehension and physical symptoms such as fatigue, heart palpitations, and tension [9]. Uncertainty about a possible future threat disrupts the ability to avoid it or to mitigate its negative impact and thus results in anxiety [10]. Stigma is usually accompanied by social discrimination and exclusion, and the direct experience of discrimination and exclusion may be enough to arouse fear and high tension, the typical symptoms of anxiety [11].

Individuals affected by obvious stigma internalize it and debase themselves as a result [12,13]. They may feel anxious for fear of discrimination and rejection from others. For example, internalized stigmatization associated with chronic infectious disease poses a psychological challenge to people living with it [11]. It can make them feel uncomfortable, cause them to miss the diagnosis, and lead to subsequent mental health problems [14]. In addition, chronic infectious disease stereotypes and stigmatization are found to be significantly positively correlated with anxiety [2]. Therefore, internalized stigmatization can cause individuals to fear rejection and abandonment. The thoughts are likely to create negative appraisals of self, leading to anxiety.

Accordingly, people who have traveled in the epicenter may have the internal belief that others will reject them when they discover the fact. The fear of discrimination and exclusion brought on by the internalized stigma of “having once been to the epicenter” makes them feel anxious.

### 2.2. The Impact of Internalized Stigmatization on Depression

Depression is a kind of negative and pessimistic mood [15]. The symptoms are mainly manifested in three aspects, including domains of mood (e.g., sadness, irritability), cognition (e.g., low self-esteem, hopelessness, and suicidal ideation), and somatic state (e.g., poor sleep quality, appetite and libido disorders, slow movement, and easily excited). According to the cognitive theory of depression, perceived low level of self-worth is a key cause of depression [16,17]. It is thus conceivable that internalized stigmatization is likely to lead to depression. When people are under the influence of internalized stigmatization, the created negative appraisals of themselves can produce some negative emotions; after maintaining this state for a long time, they are more likely to develop depression than ordinary people [18,19].

Furthermore, it has been found that people with special diseases may internalize negative stereotypes about illness and respond by internalized stigmatization [20]. They think they will suffer from social rejection and consider themselves as devalued members of society [21]. Internalized stigmatization is also expected to be positively associated with self-blame and depressive symptoms in people living with chronic infectious diseases [22]. Internalized stigmatization reflects how people living with chronic infectious diseases take in negative cognition and feelings about chronic infectious diseases and how they carry these feelings (e.g., self-deprecation, feeling dirty, self-blame) daily into interpersonal reflection and interpersonal interactions [23]. When people living with chronic infectious diseases engage in behavior toward close people (such as “taking infectious disease medications around loved ones”), their subjective and interpersonal perceptions become more negative [13]. Internalized stigmatization may prevent them from disclosing their status and seeking help from their close partners, family members, or friends. Therefore, they may be prone to depression [24].

As such, it can be supposed that the travelers who came back from the epicenter would become depressed under the influence of internalized stigmatization (e.g., believe being discriminated against or doubted by the community).

### 2.3. The Impact of Internalized Stigmatization on Shame

Shame arises when internal, stable, and global attributions about one’s self lead to negative feelings about the global self [25]. Shame strikes the core of a person’s identity and, as a result, forces the individual to contemplate the possibility of a defective, unworthy, or damaged self. The entire self is the central focus of negative evaluation [26]. Internalized stigmatization is often associated with self-deprecating emotions and cognition such as shame and self-blame [27]. People affected by internalized stigmatization blame themselves and have negative self-cognition, and they are more likely to identify with the demeaning belief [28]. It is reported that many people living with chronic infectious diseases think that they are notorious and unforgivable, and they are easily ashamed [29]. When a sense of shame arises, people tend to avoid the public [30]. The avoidance behaviors that “it is difficult to tell others about my status” and “I hide my status from others” often happen when people internalize the stigma and thus feel ashamed [8]. Accordingly, after the outbreak of the epidemic, a large number of media reports would increase the internalized stigmatization of travel experience in the epidemic area. People who came back from the epicenter may have produced self-deprecating emotions and cognition and felt ashamed as a result.

### 2.4. The Moderating Role of Social Support in the Relationship Between Internalized Stigmatization and Mental Health Problems

Social support is defined as “a social network’s provision of psychological and material resources intended to benefit an individual’s ability to cope with stress” [3]. Thus, social support involves the functional (vs. structural) operations of the broader social network. Examples of such provisions include instrumental support (e.g., provision of material or task assistance), informational support (e.g., provision of guidance that facilitates the individual’s coping or problem-solving efforts), and emotional support (e.g., provision of concern, empathy, and affection) [31].

Social support can be used as a way to regulate negative emotions. In an intimate relationship, if one partner is depressed or has other mental health problems, positive emotional and material support from the other can be effective in mitigating the negative effects [32]. Moreover, social support can enhance emotional health, which is very important to alleviate psychological distress [33]. When the level of social support is high, the negative effects of internalized stigmatization on one’s mental health can be weakened. The high level of social support means people can get more social support, such as emotional support from family and friends. This can help people affected by stigma to develop positive self-awareness and reduce negative emotions [8]. It has been found that if suspected infectious disease patients could meet with other patients and seek social support from people who have compassion for them, they could have a chance of solving the mental health problems caused by infectious diseases [34].

As such, for the travelers who came back from the epicenter, the high level of social support more likely played a key role in buffering the negative effects of internalized stigmatization on their mental health, and helped further promote the recovery of mental health in them (and vice versa, when the social support was at a low level). In other words, social support is proposed to moderate the relationship between internalized stigmatization and mental health problems (anxiety, depression, and shame) so that such a relationship is weaker when social support is high versus low.

## 3. Materials and Methods

### 3.1. Procedure

Convenience sampling was conducted in this study. The survey was conducted with the help of residential community workers who were in charge of arranging quarantine for people returning to the city of Guangzhou. The residential community workers helped us to identify those having travel experiences in Hubei Province and being currently asked to be quarantined at home to fill in the questionnaires. Considering that the residential community workers were responsible for delivering food and goods to every household of quarantined people, we asked them to help us send an invitation letter of survey to those quarantined people when delivering food and goods. The QR (two-dimension) code linking to the questionnaires was printed at the bottom of the letter. Those who were willing to participate in this survey used their smartphones to scan the QR (two-dimension) code to fill in the questionnaires online. Informed consent of participants was obtained online, and only after the “Yes, I agree” button was pressed could participants continue to fill in the questionnaire online. The questionnaires, when completed, were directly submitted online and thus were not exposed to the residential community workers. In the survey process, all the participants were told that this survey would be just used for academic research, and their answers and personal information would be kept confidential. The names, contact information, and institutions of researchers were provided in the invitation letter and online questionnaires. When in doubt, the participants could contact the researchers.

The survey used the time-lagged design to reduce common method bias, which refers to the bias in estimation resulting from the data for the predictors and outcome variables obtained from the same person in the same measurement context at the same time points [35]. Since cross-sectional data generally share the same method of collection (i.e., collected from the same person at the same points), the observed covariance between predictors and outcome variables may include covariance due to the same method [35]. Consistent with the practice used in previous studies, an effective way to avoid common method variance is to collect the data at different time points. In this study, the data were collected during two rounds. In the first round, the respondents were asked to complete measures of “internalized stigmatization” and “social support” and to report demographic information (i.e., age, gender, education). In the second round, the respondents were asked to complete measures of “anxiety”, “depression”, and “shame” and to report “how many days you have experienced being in quarantine?”. The two-round survey was conducted with a four-day interval, since the whole period of quarantine was fourteen days and a long interval between the two rounds would have caused a high sample loss rate.

As a result, among 926 respondents, 774 of them completed the two-round survey (valid response rate = 83.58%). In the sample, 415 (53.60%) were male, 86.80% of them were 20–40 years old, 97.80% of them had high school education or above, and all of them had experienced more than a four-day quarantine when they filled in the second-round questionnaire. Before they were required to be quarantined at home, they had been monitored with nucleic acid detection to ensure no obvious symptoms of the epidemic.

### 3.2. Measures

Measures of all the constructs were translated from English to Chinese through the translation–back-translation procedure (see Appendix A) [36].

Internalized stigmatization. It was measured by a 17-item scale adapted from Visser and colleagues’ measure [37]. The items were tailored to reflect the context of this study. For example, “If I was in public or private transport and someone knew I had HIV, they would not sit next to me” was rendered as “If I was in public or private transport and someone knew I had travelled in Hubei Province, they would not sit next to me”. The item parceling method was used to estimate the loading factor of this variable to deal with the estimate bias caused by too many items, and the items were sequentially parceled into four (as 4-4-4-5) to estimate the loading factor.

Social support. It was measured by a 6-item scale developed by Sarason and colleagues [38] (e.g., “You can easily find someone that you really count on to distract you from your worries when you feel under stress”).

Anxiety and depression. They were measured by a 7-item scale developed by Andrea and colleagues [39] (e.g., “Worrying thoughts go through my mind” for the measure of anxiety; “I feel as if I am slowed down”, “I feel cheerful (reverse)” for the measure of depression).

Shame. It was measured by a 6-item scale adapted from Fortenberry and colleagues’ measure [40]. The items were tailored to reflect the context of this study. For example, “Getting a sexually transmitted disease means I don’t keep myself clean” was rendered as “Travelling experience in Hubei Province means I don’t keep myself clean”.

Respondents rated the items from 1 = strongly disagree to 7 = strongly agree. Cronbach’s alpha values of scales for these variables were 0.9405, 0.898, 0.790, 0.677, and 0.885, respectively, which demonstrated that the reliability of variables in this study was adequate.

Control variables. The effects of gender, age, education levels, and time which participants had spent in quarantine (i.e., days) on individual mental health problems were controlled.

### 3.3. Confirmatory Factor Analyses

Confirmatory factor analysis was used to test if participants’ scores on the measures of constructs within this study had good convergent validity and discriminate validity. The latent variables were loaded by their respective indicators, and the correlations among the latent variables were estimated freely. Results showed that the standard factor loadings of indicators on their respective latent variables were all significant, which indicated that all the indicators corresponded well to their respective latent variables, and thus the convergent validity of constructs in this study was acceptable.

Moreover, the hypothesized five-factor model (i.e., internalized stigmatization, social support, anxiety, depression, and shame) fit the data well, χ^2^ (395, *n* = 774) = 1443.052, root mean square error of approximation (RMSEA) = 0.059, standardized residual mean root (SRMR) = 0.070, comparative fit index (CFI) = 0.911, and non-normed fit index (NNFI or TLI) = 0.902 (see Table 1). An alternative four-factor model was specified by combining internalized stigmatization and shame into one factor. This four-factor model exhibited significantly worse fit than the five-factor model; the *p*-value of Δχ^2^ (5 *n* = 774) was below 0.01, RMSEA = 0.090, SRMR = 0.329, CFI = 0.785, and TLI = 0.766. An alternative four-factor model was specified by combining constructs measured at Time 1 (i.e., internalized stigmatization and social support) into one factor. This four-factor model exhibited significantly worse fit than the five-factor model; the *p*-value of Δχ^2^ (5 *n* = 774) was below 0.01, RMSEA = 0.128, SRMR = 0.133, CFI = 0.569, and TLI = 0.532. An alternative four-factor model was specified by combining constructs measured at Time 2 (i.e., anxiety and depression) into one factor. This four-factor model also fit the data significantly worse than the five-factor model; the *p*-value of Δχ^2^ (5, *n* = 774) was below 0.01, RMSEA = 0.084, SRMR = 0.127, CFI = 0.813, and TLI = 0.797. An alternative four-factor model was specified by combining constructs measured at Time 2 (i.e., anxiety and shame) into one factor. This four-factor model also fit the data significantly worse than the five-factor model; the *p*-value of Δχ^2^ (5, *n* = 774) was below 0.01, RMSEA = 0.079, SRMR = 0.129, CFI = 0.836 and TLI = 0.822. An alternative four-factor model was specified by combining constructs measured at Time 2 (i.e., depression and shame) into one factor. This four-factor model also fit the data significantly worse than the five-factor model; the *p*-value of Δχ^2^ (5, *n* = 774) was below 0.01, RMSEA = 0.069, SRMR = 0.086, CFI = 0.874 and TLI = 0.863. An alternative three-factor model was specified by combining all the constructs measured at Time 2 (i.e., anxiety, depression, and shame) into one factor. This three-factor model also fit the data significantly worse than the five-factor model; the *p*-value of Δχ^2^ (9, *n* = 774) was below 0.01, RMSEA = 0.094, SRMR = 0.125, CFI = 0.766 and TLI = 0.748. As such, the above comparison of measurement models demonstrated that the discriminate validity of constructs in this study was adequate.

### 3.4. Analytic Strategy

The study presented a moderation model. The hypothesized relationships of variables were simultaneously estimated using the structural equation modeling (SEM) method in Mplus 7.0 (Muthén & Muthén, Los Angeles, CA, USA) [41]. For interpretation purposes, gender, age, education, days, stigmatization, and social support were all grand-mean centered. In addition, the confirmatory factor analysis was estimated using Mplus 7.0, while the reliability test and descriptive statistical analysis were conducted using SPSS 16.0 (IBM, Armonk, NY, USA).

## 4. Results

Means, standard deviations, and bivariate correlations of variables in the study are shown in Table 2. Stigmatization was positively correlated with anxiety, depression, and shame (*r* = 0.274, 0.272, and 0.248, *p* < 0.01, respectively). These findings provided preliminary support for the hypothesized relationships.

Table 3 shows the results of the hierarchical multiple regression analyses that tested the effects of stigmatization on anxiety, depression, and shame, and the moderation effects of social support on relationships between stigmatization and anxiety, depression, and shame. As shown in Table 3, stigmatization positively predicted anxiety, depression, and shame (*b* = 0.257, 0.149, and 0.114, *p* < 0.01 in Step 2). Moreover, the results of the interaction between stigmatization and social support on anxiety, depression, and shame demonstrated the negative effects of social support on the slopes between stigmatization and anxiety, depression, and shame (*b* = −0.222, −0.133, and −0.106, *p* < 0.01 in Step 3).

**Figure 1:**
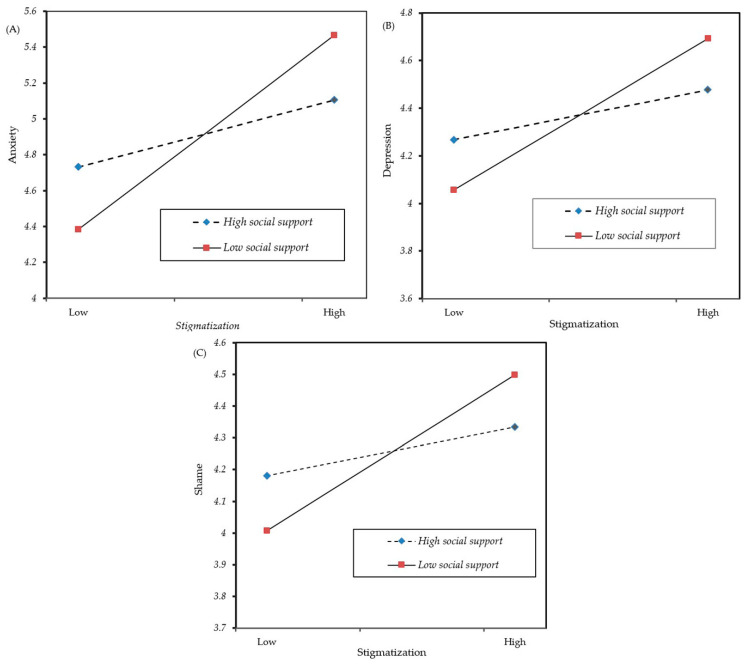
The moderating role of social support in the relation between stigmatization of epicenter travel experience and anxiety (**A**), depression (**B**) and shame (**C**).

Table 4 shows the results of relationships between stigmatization and anxiety, depression, and shame at high (+1 SD) and low level (−1 SD) of social support. The results showed that the effects of stigmatization on anxiety, depression, and shame were significantly weaker at high-level (+1 *SD*) social support (*Estimate* = 0.134, 0.075, and 0.055, *p* < 0.01, 0.01, and 0.05) than that at low-level (−1 *SD*) social support (*Estimate* = 0.390, 0.229, and 0.177, *p* < 0.01; *z* = −0.257, −0.154, −0.122, *p* < 0.01). The analysis results above were also displayed in Figure 1. Using Cohen, Cohen, West, and Aiken’s (2003) method [42], we plotted the interaction at high (+1 SD) and low level (−1 SD) of social support. As shown in Figure 1, when social support was higher, the relationships between stigmatization and anxiety, depression, and shame were weaker.

## 5. Discussion

The empirical findings show that the internalized stigmatization has positive relationships with mental health problems (including anxiety, depression, and shame) for people who once traveled in the epicenter. The internalized stigmatization associated with travel experiences in the epicenter is also worth paying attention to, compared with that brought on by the physical status of having an infectious disease. Since the novel coronavirus disease has rapidly spread to various provinces of China and has caused a great many deaths, this serious situation is bound to cause a national psychological panic. Therefore, under the influence of the severe public opinion environment, the discrimination suffered by the respondents who had traveled to Hubei will be more direct and acute.

The psychological impact of internalized stigmatization remains a constant state with the epidemic that has lasted for months. However, it is comforting to find in this study that social support can play an important role in buffering the negative effects of internalized stigmatization on mental health. During the epidemic, social support (material or emotional) provided by the family and the community is shown to be effective in helping people with travel experiences in the epicenter to get through the quarantine period.

We have not observed the significant independent effect of social support on mental health problems. Prior studies pay attention to social support as the important moderation in alleviating negative psychological process. Social support is a construct with the comparatively long-term implication. The perceived social support reflects the social cohesion that is gradually formed in a long period and in the process of continued social interaction among individuals [43]. In contrast, the anxiety, depression, and shame, in some cases, can be regarded as a comparatively short-term or event-contingent construct. For example, in the background of this study, anxiety, depression, and shame are stigmatization-aroused. When such strong stimuli gradually recede, the above mental health problems can be relieved. Therefore, it is not surprising to find that people who have a higher level of social support can still encounter possible mental health problems when faced with adverse life experience. Besides, the direct and independent effect of social support on negative psychological problems (e.g., depression) remains undetermined. Although some studies believe that social support as an independent factor may alleviate the depression symptom, there are a few studies argue that individuals with strong social support (many friends or social cohesion) can still be easier to suffer from depression symptoms, as the efforts to maintain strong social support would also bring extra psychological burden [44].

This study has some theoretical implications. Theoretically, previous studies mainly focused on the negative influence of stigmatization suffered by patients living with chronic infectious diseases. The stigmatization suffered by people who are not patients, but who are implicated in infectious diseases, has been given less attention. Moreover, previous studies paid much attention to the attributed stigmatization reflecting how most of people perceive a stigma, but did not explore the internalized stigmatization that indicates how respondents feel about stigmas suffered by themselves. As such, this study fills in the research gap by helping one to understand the negative psychological reactions of people who suffer internalized stigmatization brought on by travel experiences in the epicenter after the outbreak of pandemic. The findings of this study support the hypotheses that internalized stigmatization can also have a negative impact on the mental health of people who have traveled in Hubei Province but did not necessarily get infected. These people have reported higher levels of anxiety, depression, and shame due to the internalized stigma of travel experiences in the epicenter. However, this does not mean that such negative effects cannot be eliminated. The empirical results of this study also reveal the underlying moderation mechanism, suggesting that although internalized stigmatization is detrimental to mental health, a high level of social support can effectively regulate and weaken such negative effects.

This study has some practical implications. On the one hand, the findings can help those with travel experiences in the epicenter to realize the underlying psychological process resulting in their mental health problems, and provide scientific evidence to doctors for formulating targeted psychotherapy programs. On the other hand, this study shows that social support has a regulatory role, which can remind people, suffering internalized stigmatization in the epidemic, to seek social support (e.g., seeking help from family, friends, or even psychologists to better solve mental health problems). At the same time, the community, social institutions, and government departments can also help this group of people in time, such as by arranging psychological counseling based on the actual condition, to prevent their mental health problems from further development.

However, this study is not free of limitations. First, in order to avoid common method bias from cross-sectional data, the present study used the time-lagged design to collect data at different time points, but the conclusions drawn from this design still did not completely reflect causality. Hence, future research should conduct the experimental design or use longitudinal data to ensure that the conclusion reflects causality.

Second, limited by human, material, and financial resources as well as other factors, the present study adopted the convenience sampling method to collect data rather than random sampling. Because the convenience sample may not be enough to represent the population of interest, the findings of this study may, to some extent, only apply to the population. Future research can use additional samples to investigate the validity and transportability of our findings.

Third, future research can discuss the relevant issues happening to different groups of people. For example, the medical staff who take responsibility for taking care of infected patients are more likely to suffer the stigma when they return to their life domain. Future research can also carry out fine-grained analysis among different groups suffering the stigma (such as healthy people with certain racial or ethnic characteristics of susceptible population, once infected people, and medical staff) or among different regions and countries with a large-scale outbreak of this epidemic.

## 6. Conclusions

This study finds that after the outbreak of the epidemic in Hubei Province, people with travel experiences in the epicenter were affected by internalized stigmatization, and thus suffered anxiety, depression, and shame. Social support was also found to be an effective way to weaken the negative influence of internalized stigmatization. When the level of social support is high, the negative impact of internalized stigmatization on people’s mental health is shown to be weaker, whereas when social support is at a low level, such negative impact appears stronger. The provision of much more social support can serve as an important solution to alleviating the mental health problems of people suffering from internalized stigmatization due to their epicenter travel experiences.

## Figures and Tables

**Table 1:** Comparison of measurement models.

Model	Description	χ^2^	*df*	Δχ^2^	RMSEA	SRMR	CFI	TLI
The baseline five-factor model	ST, SS, AN, DE, SH.	1443.052	395		0.059	0.070	0.911	0.902
The four-factor model ^a^	ST and SH were combined into one factor.	2932.681	400	1489.629 **	0.090	0.329	0.785	0.766
The four-factor model	ST and SS were combined into one factor.	5469.909	400	4026.857 **	0.128	0.133	0.569	0.532
The four-factor model	AN and DE were combined into one factor.	2599.756	400	1156.704 **	0.084	0.127	0.813	0.797
The four-factor model	AN and SH were combined into one factor.	2327.843	400	884.791 **	0.079	0.129	0.836	0.822
The four-factor model	DE and SH were combined into one factor.	1886.997	400	443.945 **	0.069	0.086	0.874	0.863
The three-factor model	AN, DE, and SH were combined into one factor.	3163.953	404	1720.901 **	0.094	0.125	0.766	0.748

Note: ST = internalized stigmatization; SS = social support; AN = anxiety; DE = depression; SH = shame. RMSEA = root mean square error of approximation; SRMR = standardized residual mean root; CFI = comparative fit index; TLI = non-normed fit index (NNFI). ** *p* < 0.01. ^a^ Consistent with the practice used in previous studies, the comparison of measurement models is conducted based on the fact that the time-lag design ensured the acceptable discriminate validity of variables measured at different time points. Thus, the comparison of measurement models focuses on the test of discriminate validity of variables measured at the same time point (at Time 1 or Time 2 for this study). Even so, the test of discriminate validity of internalized stigmatization and shame, suggested by the reviewer, had to be conducted. Thus, an alternative four-factor model that combined internalized stigmatization and shame into one factor was used to compare with the baseline five-factor model.

**Table 2:** Means, standard deviations, and bivariate correlations among variables.

Variable	M	SD	1	2	3	4	5	6	7	8	9
1. Gender	0.460	0.499									
2. Age	32.940	5.959	0.050								
3. Education	15.800	1.560	0.001	−0.010							
4. Days	7.360	1.945	−0.018	−0.092 *	−0.060						
5. Stigmatization	4.582	0.694	−0.025	−0.055	0.028	−0.017	(0.945)				
6. Social support	3.386	0.577	0.076 *	0.051	−0.016	−0.003	0.076 *	(0.898)			
7. Anxiety	4.914	0.640	0.041	0.030	0.028	0.053	0.274 **	0.014	(0.790)		
8. Depression	4.369	0.376	0.073 *	−0.026	0.006	0.055	0.272 **	0.016	0.365 **	(0.677)	
9. Shame	4.252	0.315	0.046	−0.051	−0.048	0.050	0.248 **	0.021	0.290 **	0.276 **	(0.885)

Note: *n* = 774. Gender is coded as 0 for males and 1 for females. Internal consistency coefficients, Cronbach’s alphas are reported in the parentheses on the diagonal. * p < 0.05, ** *p* < 0.01.

**Table 3:** Regression results using structural equation modeling (SEM).

Variable	Regression 1. Dependent variable: Anxiety		
	Step 1	Step 2	Step 3
**Demographics**			
Gender	0.051 (0.045)	0.059 (0.044)	0.062 (0.044)
Age	0.004 (0.004)	0.005 (0.004)	0.005 (0.004)
Education	0.013 (0.013)	0.010 (0.013)	0.011 (0.013)
Days	0.019 (0.011)	0.021 * (0.011)	0.020 (0.011)
**Independent variable**			
Stigmatization		0.257 ** (0.026)	0.262 ** (0.026)
**Moderator**			
Social Support			−0.005 (0.035)
**Interaction**			
Stigmatization × Social Support			−0.222 ** (0.049)
	**Regression 2. Dependent variable: Depression**		
	**Step 1**	**Step 2**	**Step 3**
**Demographics**			
Gender	0.056 * (0.027)	0.061 * (0.026)	0.062 * (0.026)
Age	−0.002 (0.002)	−0.001 (0.002)	−0.001 (0.002)
Education	0.002 (0.009)	0.001 (0.008)	0.001 (0.008)
Days	0.011 (0.007)	0.012 (0.007)	0.011 (0.007)
**Independent variable**			
Stigmatization		0.149 ** (0.016)	0.152 ** (0.016)
**Moderator**			
Social Support			−0.002 (0.020)
**Interaction**			
Stigmatization × Social Support			−0.133 ** (0.031)
	**Regression 3. Dependent variable: Shame**		
	**Step 1**	**Step 2**	**Step 3**
**Demographics**			
Gender	0.031 (0.023)	0.034 (0.023)	0.035 (0.023)
Age	−0.003 (0.002)	−0.002 (0.002)	−0.002 (0.002)
Education	−0.009 (0.007)	−0.011 (0.008)	−0.010 (0.007)
Days	0.007 (0.005)	0.008 (0.005)	0.007 (0.005)
**Independent variable**			
Stigmatization		0.114 ** (0.017)	0.116 ** (0.016)
**Moderator**			
Social Support			0.004 (0.018)
**Interaction**			
Stigmatization × Social Support			−0.106 ** (0.032)

Note: *n* = 774. Value are unstandardized regression coefficients; standard error estimates are in parentheses. * *p* < 0.05, ** *p* < 0.01.

**Table 4:** Estimates for moderation effect of social support.

Variable	P_Y1X_		P_Y2X_		P_Y3X_	
	β	S.E.	β	S.E.	β	S.E.
Zero Social Support	0.257 **	0.026	0.149 **	0.016	0.114 **	0.017
High Social Support (+1 SD)	0.134 **	0.037	0.075 **	0.021	0.055 *	0.026
Low Social Support (−1 SD)	0.390 **	0.039	0.229 **	0.026	0.177 **	0.024
High- and Low-Level Difference z	−0.257 **	0.056	−0.154 **	0.035	−0.122 **	0.037

Note: *n* = 774. Value are unstandardized regression coefficients. *P_Y1X_* is the path from stigmatization to anxiety; *P_Y2X_* is the path from stigmatization to depression; *P_Y3X_* is the path from stigmatization to shame. * p < 0.05, ** p < 0.01. S.E. = standard error.

## Appendix A

**Table A1:** The scales of constructs.

Internalized Stigmatization (1 = Strongly Disagree to 7 = Strongly Agree) [37]
1. Having travelled in Hubei Province is a punishment for bad behavior.
2. If I was in public or private transport and someone knew I had travelled in Hubei Province, they would not sit next to me.
3. I think having travelled in Hubei Province was just a matter of bad luck.
4. I think less of myself because I have travelled in Hubei Province.
5. My neighbors would not like me living next door if they knew I had travelled in Hubei Province.
6. I would understand if people rejected my friendship because I have travelled in Hubei Province.
7. I feel it is completely safe for me to handle other people’s children (reverse).
8. I have a lot to teach people about life through having travelled in Hubei Province (reverse).
9. Because of my travelling experience in Hubei Province, people would not date me.
10. People are right to be afraid of me because I have travelled in Hubei Province.
11. I feel that it is my fault that I have travelled in Hubei Province.
12. Although I have travelled in Hubei Province, I am a person who deserves as much respect as anyone else.
13. Most employers would not employ me because I have travelled in Hubei Province.
14. If I drank from a tap and people knew I had travelled in Hubei Province, they would not drink from the same tap.
15. I must have done something wrong to deserve having travelled in Hubei Province.
16. I feel ashamed that I have travelled in Hubei Province.
17. When people know I have travelled in Hubei Province, I feel uncomfortable around them.
**Anxiety (1 = Strongly Disagree to 7 = Strongly Agree) [39]**
1. I feel tense or “wound up”.
2. I get a sort of frightened feeling as if something awful is about to happen.
3. Worrying thoughts go through my mind.
4. I can sit at ease and feel relaxed.
5. I get a sort of frightened feeling like “butterflies” in my stomach.
6. I feel restless as if I have to be on the move.
7. I get sudden feelings of panic.
**Depression (1 = Strongly Disagree to 7 = Strongly Agree) [39]**
1. I still enjoy the things I used to enjoy (reverse).
2. I can laugh and see the funny side of things (reverse).
3. I feel cheerful (reverse).
4. I feel as if I am slowed down.
5. I have lost interest in my appearance.
6. I look forward with enjoyment to things (reverse).
7. I can enjoy a good book or radio or TV program (reverse).
**Shame (1 = Strongly Disagree to 7 = Strongly Agree)** [40]
1. Getting a travelling experience in Hubei Province means a person has been hanging with the wrong crowd.
2. A travelling experience in Hubei Province means I don’t keep myself clean.
3. Getting a travelling experience in Hubei Province means a person should be ashamed of himself or herself.
4. Getting a travelling experience in Hubei Province means a person is dirty.
5. Getting a travelling experience in Hubei Province means I don’t take care of myself.
6. Getting examined for a travelling experience in Hubei Province means I’m not clean.
**Social support questionnaire (SSQ-6, 1 = Strongly Disagree to 7 = Strongly Agree).** [38]
1. You can easily find someone that you really count on to distract you from your worries when you feel under stress.
2. You can easily find someone that you really count on to help you feel more relaxed when you are under pressure or tense.
3. You can easily find someone that accepts you totally, including both your worst and your best points.
4. You can easily find someone that you really count on to care about you, regardless of what is happening to you.
5. You can easily find someone that you really count on to help you feel better when you are generally feeling down in the dumps.
6. You can easily find someone that you count on to console you when you are very upset.
